# Comparison of vildagliptin twice daily vs. sitagliptin once daily using continuous glucose monitoring (CGM): Crossover pilot study (J-VICTORIA study)

**DOI:** 10.1186/1475-2840-11-92

**Published:** 2012-08-06

**Authors:** Masaya Sakamoto, Rimei Nishimura, Taiga Irako, Daisuke Tsujino, Kiyotaka Ando, Kazunori Utsunomiya

**Affiliations:** 1 Division of Diabetes, Metabolism and Endocrinology, Department of Internal Medicine, Jikei University School of Medicine, 3-25-8 Nishi-Shinbashi, Minato-ku, Tokyo, 105-8461, Japan; 2 Graduate School of Public Health, University of Pittsburgh, Pittsburgh, Pennsylvania, USA

**Keywords:** Vildagliptin, Sitagliptin, Continuous glucose monitoring (CGM), Brain natriuretic peptide (BNP), Plasminogen activator inhibitor-1 (PAI-1)

## Abstract

**Background:**

No previous studies have compared the DPP-4 inhibitors vildagliptin and sitagliptin in terms of blood glucose levels using continuous glucose monitoring (CGM) and cardiovascular parameters.

**Methods:**

Twenty patients with type 2 diabetes mellitus were randomly allocated to groups who received vildagliptin then sitagliptin, or vice versa. Patients were hospitalized at 1 month after starting each drug, and CGM was used to determine: 1) mean (± standard deviation) 24-hour blood glucose level, 2) mean amplitude of glycemic excursions (MAGE), 3) fasting blood glucose level, 4) highest postprandial blood glucose level and time, 5) increase in blood glucose level after each meal, 6) area under the curve (AUC) for blood glucose level ≥180 mg/dL within 3 hours after each meal, and 7) area over the curve (AOC) for daily blood glucose level <70 mg/dL. Plasma glycosylated hemoglobin (HbA1c), glycoalbumin (GA), 1,5-anhydroglucitol (1,5AG), immunoreactive insulin (IRI), C-peptide immunoreactivity (CPR), brain natriuretic peptide (BNP), and plasminogen activator inhibitor-1 (PAI-1) levels, and urinary CPR levels, were measured.

**Results:**

The mean 24-hour blood glucose level was significantly lower in patients taking vildagliptin than sitagliptin (142.1 ± 35.5 vs. 153.2 ± 37.0 mg/dL; p = 0.012). In patients taking vildagliptin, MAGE was significantly lower (110.5 ± 33.5 vs. 129.4 ± 45.1 mg/dL; p = 0.040), the highest blood glucose level after supper was significantly lower (206.1 ± 40.2 vs. 223.2 ± 43.5 mg/dL; p = 0.015), the AUC (≥180 mg/dL) within 3 h was significantly lower after breakfast (484.3 vs. 897.9 mg/min/dL; p = 0.025), and urinary CPR level was significantly higher (97.0 ± 41.6 vs. 85.2 ± 39.9 μg/day; p = 0.008) than in patients taking sitagliptin. There were no significant differences in plasma HbA1c, GA, 1,5AG, IRI, CPR, BNP, or PAI-1 levels between patients taking vildagliptin and sitagliptin.

**Conclusions:**

CGM showed that mean 24-h blood glucose, MAGE, highest blood glucose level after supper, and hyperglycemia after breakfast were significantly lower in patients with type 2 diabetes mellitus taking vildagliptin than those taking sitagliptin. There were no significant differences in BNP and PAI-1 levels between patients taking vildagliptin and sitagliptin.

**Trial registration:**

UMIN000007687

## Introduction

The number of patients with type 2 diabetes mellitus is rapidly increasing worldwide, especially in Asian countries, because of an aging population and changes in dietary habits. The management of blood glucose levels has become a significant medical issue. The short-term aim of diabetes treatment is control of blood glucose levels, and the long-term aim is avoidance of the complications of diabetes [1].

Glycosylated hemoglobin (HbA1c) level reflects the blood glucose level over the preceding 2 months, and can be used to diagnose diabetes or to evaluate blood glucose control in patients known to be diabetic. Clinical and observational studies have reported that reducing HbA1c levels results in a lower incidence of cardiovascular complications in diabetic patients with a shorter time since diagnosis [2,3], but not in diabetic patients with a longer time since diagnosis [4,5]. Starting treatment for diabetes at an earlier stage is therefore thought to be important for decreasing the risk of cardiovascular events.

Current diabetes treatment programs aim to lower HbA1c levels. However, recent clinical studies have found that hypoglycemia and postprandial hyperglycemia are also associated with the development of cardiovascular disease [6,7]. Treatment choices should therefore consider control of variations in blood glucose levels, as well as HbA1c levels, to reduce the risk of cardiovascular events. Continuous glucose monitoring (CGM) and self-monitoring of blood glucose routinely record variations in blood glucose levels [8]. CGM can evaluate changes in blood glucose levels, because it allows recording over several days.

DPP-4 inhibitors are oral antihyperglycemic drugs that have recently become available for diabetes treatment. They enhance the actions of incretin, which promotes insulin secretion and suppresses glucagon secretion depending on blood glucose levels [9], thereby improving blood glucose control without inducing hypoglycemia. Various effects of incretin such as pancreatic β-cell protection and cardiovascular protection [10] are expected to reduce the risk of development of cardiovascular diseases. DPP-4 inhibitors are considered effective for the treatment of type 2 diabetes mellitus in Asian patients, including Japanese patients, who often have insufficient insulin secretion [11,12], in contrast to Caucasian patients who usually have insulin resistance. However, few studies have examined differences in the control of blood glucose levels between different DPP-4 inhibitors. Sitagliptin and vildagliptin are known to have different efficacy in suppressing DPP-4 activity. We conducted a crossover pilot study named Jikei-Vildagliptin and sitagliptin with CGM TO Real blood glucose control in type 2 diAbetes (J-VICTORIA). This study compared the DPP-4 inhibitors vildagliptin and sitagliptin, using CGM to evaluate blood glucose levels and analyze fluctuations in blood glucose levels. We also compared cardiovascular parameters between patients taking vildagliptin and sitagliptin by measuring plasma levels of brain natriuretic peptide (BNP) and plasminogen activator inhibitor-1 (PAI-1).

## Subjects and methods

### Subjects

Patients with type 2 diabetes mellitus who had poor control of blood glucose levels (HbA1c 6.5–9.5%) in spite of diet and exercise therapy for 1 month or longer, with or without oral antidiabetic treatment, were included in the study. Exclusion criteria were: 1) type 1 diabetes mellitus, 2) severe ketosis, coma, or reduced level of consciousness due to diabetes within the past 6 months, 3) severe infection, pre- or postoperative, or severe trauma, 4) history of laparotomy or ileus, 5) chronic intestinal disease associated with a disorder of digestion or absorption, 6) severe hernia, or stenosis or ulcer of the large intestine, 7) pregnancy, possible pregnancy, or breastfeeding, 8) moderate or severe renal dysfunction (creatinine clearance <50 mL/min, serum creatinine level ≥1.5 mg/dL in men or ≥1.3 mg/dL in women), 9) severe hepatic dysfunction, 10) insulin treatment, 11) treatment with antidiabetic agents other than sulfonylureas, 12) history of hypersensitivity to any of the ingredients of the study drugs and 13) judged to be unsuitable for participation for medical reasons. Patients were given detailed explanations of the study protocol. Those who provided informed consent were included in the study. The study protocol was approved by the Ethical Committee of the Jikei University School of Medicine. The Clinical Trial registration No. is UMIN000007687.

## Methods

**Figure 1:**
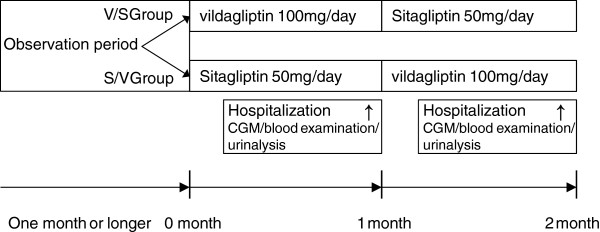
**Study protocol.** After an outpatient observation period of 1 month or longer, patients were randomized to the V/S group which initially received vildagliptin (50 mg twice daily) or the S/V group which initially received sitagliptin (50 mg once daily). The drugs were then switched so that the V/S group received sitagliptin and the S/V group received vildagliptin. **↑** indicates continuous glucose monitoring (CGM) on the second and third hospital days. Identical meals were given to all patients during hospitalization.

Figure 1 shows a summary of the study protocol. Patients were randomly allocated to the V/S or S/V group at the beginning of study period. During Stage I, the V/S group received vildagliptin 100 mg daily (50 mg in the morning and 50 mg in the evening) and the S/V group received sitagliptin 50 mg daily (in the morning). This reflects the normal doses of these drugs used in Japan. After 1 month of treatment, patients were hospitalized for 4 days, and blood glucose levels were measured for two consecutive days using CGM (CGMS-gold; Medtronic Minimed, Northridge, CA, USA). During Stage II, the V/S group received sitagliptin (50 mg daily) and the S/V group received vildagliptin (50 mg twice daily). After 1 month of Stage II treatment, subjects were hospitalized for another 4 days to measure blood glucose levels. Administration of other antihyperglycemic drugs was prohibited during the study period.

The followings values were calculated using CGM data: 1) mean 24-h (± standard deviation) blood glucose level, 2) mean amplitude of glycemic excursions (MAGE), 3) fasting blood glucose level, 4) highest postprandial blood glucose level and time, 5) increase in blood glucose level after each meal, 6) area under the curve (AUC) for blood glucose level ≥180 mg/dL within 3 h after each meal, and 7) area over the curve (AOC) for daily blood glucose level <70 mg/dL.

Other data collected were plasma HbA1c, glycoalbumin (GA), 1,5-anhydroglucitol (1,5AG), immunoreactive insulin (IRI), C-peptide immunoreactivity (CPR), BNP, PAI-1, and 24-h urinary CPR levels. The subjects ingested identical meals during hospitalization, and were advised not to change their level of exercise.

### Statistical analysis

Data are shown as the mean ± standard deviation. The paired *t*-test was used to compare values between patients taking different drugs, with the level of significance set at p < 0.05. Statistical analysis of data was performed using the Statistical Package for Social Sciences software, version 19.0 (SPSS, Chicago, IL, USA).

## Results

A total of 20 patients were enrolled in the study, with a mean age of 55.2 ± 15.5 years, mean body mass index of 25.1 ± 5.4 kg/m^2^, mean HbA1c level of 7.9 ± 0.7%, and mean time since diagnosis of diabetes of 4.5 ± 3.7 years. Before enrollment in the study, 8 patients were taking a DPP-4 inhibitor in combination with a sulfonylurea and 12 were taking a DPP-4 inhibitor only (Table 1). The mean time since diagnosis of diabetes was relatively short (4.5 years). None of the patients had clinical stage IV peripheral arterial occlusive disease or lower limb amputation.

**Table 1:** Baseline patient characteristics

**Characteristic**	**Value**
Age (years)	55.2 ± 15.5
Gender (M/F)	13/7
Body Mass Index (Kg/m2)	25.1 ± 5.4
Diabetes Duration (years)	4.5 ± 3.7
HbA1c (%)	7.9 ± 0.7
Treatment	
None (diet/exercise)	12
SU	8

HbA1c = glycosylated hemoglobin; SU = sulfonylurea.

**Figure 2:**
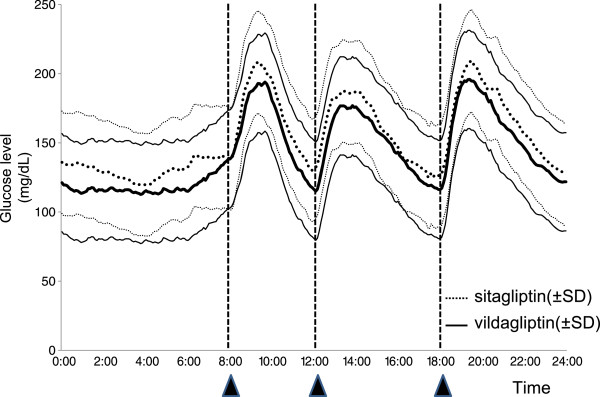
**Glucose levels over 24 h during treatment with vildagliptin or sitagliptin in 20 patients.** Data are mean ± standard deviation.

Variations in 24-h blood glucose levels measured by CGM during treatment with sitagliptin and vildagliptin are shown in Figure 2, and blood glucose indexes derived from CGM results are shown in Table 2.

**Table 2:** Parameters of glucose variability in patients taking vildagliptin (100 mg daily) or sitagliptin (50 mg daily)

	**Vildagliptin**	**Sitagliptin**	**P-value**
24-h mean glucose level (mg/dL)	142.1 ± 14.0	153.2 ± 29.7	0.012*
0:00 to 08:00 (night) mean glucose level (mg/dL)	117.4 ± 22.1	130.9 ± 26.3	0.042*
08:00 to 24:00 (day) mean glucose level (mg/dL)	154.1 ± 25.6	164.4 ± 35.3	0.043*
SD over 24 h (mg/dL)	35.5 ± 12.6	37.0 ± 13.9	0.542
Preprandial glucose level (mg/dL)	110.5 ± 33.5	129.4 ± 45.1	0.040*
Preprandial glucose level (mg/dL)	128.1 ± 16.2	133.5 ± 26.8	0.282
lunch	109.1 ± 22.2	120.9 ± 44.8	0.165
Highest postprandial glucose level within 3 hours after each meal (mg/dL)	112.4 ± 21.1	116.6 ± 24.7	0.221
Highest postprandial glucose level within 3 hours after each meal (mg/dL)	211.0 ± 40.0	228.0 ± 58.4	0.117
lunch	188.6 ±37.7	203.1 ± 50.3	0.172
supper	206.1 ± 40.2	223.2 ± 43.5	0.015*
Time from start of meal to the highest postprandial glucose level (minutes)			
breakfast	76.0 ± 18.0	86.0 ± 28.4	0.204
lunch	93.3 ± 30.2	99.0 ± 35.6	0.579
supper	81.8 ± 24.6	93.0 ± 26.4	0.223
Differences between preprandial and highest postprandial glucose level for each meal (mg/dL)			
breakfast	83.0 ± 37.1	94.5 ± 46.3	0.185
lunch	79.5 ± 31.8	82.3 ± 33.9	0.774
supper	93.7 ± 35.7	106.6 ± 43.2	0.065
AUC (≧180 mg/dL) for glycemic variability within 3 h of each meal (mg·min/dL)			
breakfast	484.3 ± 541.1	897.9 ± 1097	0.025*
lunch	306.0 ± 554.8	630.5 ± 1017.2	0.152
supper	523.5 ± 618.3	703.4 ± 676.4	0.106
AOC (<70 mg·/dL) for glycemic variability in 24 h (mg·min/dL)			
	95.6 ± 243.0	16.2 ± 61.2	0.183

Data are mean ± standard deviation. Paired-sample *t*-test. * p < 0.05.

AUC = area under the curve; AOC = area over the curve.

The mean 24-h blood glucose level was significantly lower in patients taking vildagliptin than patients taking sitagliptin (p = 0.012) during both the night (00:00 to 08:00) and the day (08:00 to 24:00). The standard deviation of blood glucose levels was lower in patients taking vildagliptin than patients taking sitagliptin, but this difference was not significant. MAGE was significantly lower in patients taking vildagliptin than patients taking sitagliptin (p = 0.040). Although there was no significant difference in preprandial glucose levels between drugs, both the highest postprandial glucose level within 3 h (peak value) and the amplitude of increase in glucose level (the difference between the preprandial value and the peak value within 3 h) were lower in patients taking vildagliptin than patients taking sitagliptin after each meal. The peak value after dinner was significantly lower in patients taking vildagliptin than patients taking sitagliptin (p = 0.015). There was no difference in the time taken to reach the highest postprandial glucose level between patients taking vildagliptin and patients taking sitagliptin.

The AUC (>180 mg/dL) within 3 h was smaller after each of breakfast, lunch, and dinner in patients taking vildagliptin than patients taking sitagliptin, and this difference was significant after breakfast (p = 0.025). The AOC (<70 mg/dL) was comparable for vildagliptin (95.6 mg/min/dL) and sitagliptin (16.2 mg·min/dL).

There were no differences between in plasma HbA1c, GA, and 1,5-AG levels, which are indexes of blood glucose control, between patients taking vildagliptin and patients taking sitagliptin. There was no significant change in mean body weight in either group during the study period.

There were also no differences in plasma BNP, PAI-1, IRI, or CPR levels between patients taking vildagliptin and patients taking sitagliptin, but the urinary CPR level was significantly higher in patients taking vildagliptin than patients taking sitagliptin (p = 0.008) (Table 3).

**Table 3:** Glucose and cardiovascular parameters in patients taking vildagliptin (100 mg daily) or sitagliptin (50 mg daily)

	**vildagliptin**	**sitagliptin**	**p-value**
HbA1c (%)	7.54 ± 0.93	7.64 ± 0.93	0.211
GA (%)	19.8 ± 3.47	20.1 ± 3.31	0.087
1,5AG (μg/mL)	7.68 ± 5.60	7.49 ± 6.16	0.389
IRI (μU/mL)	7.26 ± 4.44	6.94 ± 4.37	0.735
CPR (ng/mL)	2.14 ± 0.92	1.95 ± 0.78	0.302
U-CPR (μ/day)	97.0 ± 41.6	85.2 ± 39.9	0.008*
BNP (pg/mL)	7.00 ± 9.24	8.03 ± 8.40	0.283
PAI-1 (ng/mL)	36.6 ± 14.9	40.6 ± 23.1	0.231

GA = glycoalbumin; 1,5-AG = 1,5-anhydroglucitol; IRI = immunoreactive insulin; CPR = C-peptide immunoreactivity; BNP = brain natriuretic peptide; PAI-1 = plasminogen activator inhibitor-1.

Data are mean ± standard deviation. Paired-sample *t*-test. * p < 0.05.

## Discussion

The influence of the DPP-4 inhibitors vildagliptin (100 mg daily) and sitagliptin (50 mg daily) on blood glucose levels in patients with type 2 diabetes mellitus was investigated in this crossover study. Mean 24-h blood glucose level and MAGE were significantly lower in patients taking vildagliptin than patients taking sitagliptin.

Differences in drug efficacy between vildagliptin and sitagliptin have been investigated in a few studies. A comparison of the randomized trials conducted in Japan showed that vildagliptin 100 mg daily resulted in lower HbA1c levels (by approximately 0.3%) than sitagliptin 50 mg daily [13]. A meta-analysis of studies using doses between 50 mg and 100 mg for both drugs found that the efficacy of vildagliptin and sitagliptin were comparable [14,15].

Kishimoto et al. reported that sitagliptin effectively reduced postprandial blood glucose fluctuations and stabilized blood glucose levels [16]. Sitagliptin may also work as a vasoprotective agent in diabetes by blocking the AGE-RAGE axis [17]. Marfella et al. compared vildagliptin (100 mg daily) and sitagliptin (100 mg daily) using CGM, and reported a better MAGE in the vildagliptin group, although there was no difference in mean 24-h blood glucose level between the groups [18]. Rizzo et al. reported recently that reductions in oxidative stress and markers of systemic inflammation were greater in patients with type 2 diabetes taking vildagliptin than those taking sitagliptin [19]. These results were obtained in Caucasian patients, and it important to also compare the effects of these drugs in Asian patients, including Japanese patients, because there are differences in causes of diabetes, insulin secretion, and background characteristics between Caucasian and Japanese patients.

The reason for the differences in drug efficacy (mean blood glucose level and MAGE) observed in this study is considered to be that sitagliptin 50 mg daily results in less than 70% suppression of DPP-4 activity over 24 h [20] whereas vildagliptin 50 mg twice daily results in 80% or greater suppression of DPP-4 activity over 24 h [21]. It is also possible that the different mode of binding with DPP-4 and the different frequency of drug administration results in a greater reduction in blood glucose level after supper and breakfast in patients taking vildagliptin [22].

For patients taking vildagliptin it is possible that suppression of glucagon secretion by the evening dose leads to a lower AUC (≥180 mg/dL) after breakfast, and suppression of glucagon secretion by the morning dose leads to a lower peak in blood glucose level after supper. These results seem to support a twice daily administration schedule for vildagliptin.

Postprandial hyperglycemia has been reported to trigger vascular disorders and cause cardiovascular events, and is more common in patients with high HbA1c levels [23]. Selection of a DPP-4 inhibitor that effectively suppresses postprandial hyperglycemia contributes to the maintenance of ideal HbA1c levels.

It is interesting that urinary CPR level and variations in blood glucose level were higher in patients taking vildagliptin than patients taking sitagliptin. We postulate that the higher level of DPP-4 inhibition over 24 h in patients taking vildagliptin, compared with patients taking sitagliptin, inhibits the destruction of incretin, which enhances endogenous insulin secretion, thereby improving MAGE and achieving a more stable reduction in blood glucose levels. A relationship between MAGE and oxidation stress has also been reported [24], suggesting that improvement of MAGE by vildagliptin administration might suppress oxidation stress and decrease the incidence of adverse cardiovascular events in the long term. This possibility should be further elucidated in future studies.

As DPP-4 inhibitors are reported to lower BNP level [25], and vildagliptin is reported to suppress PAI-1 production [26], this study measured BNP and PAI-1 levels to evaluate cardiovascular parameters, but no differences were observed between patients taking vildagliptin and sitagliptin. Linagliptin, other DPP-4 inhibitor, has been reported to be beneficial in terms of cardiac protection and safety [27]. Improvements in parameters might be due to the effects of the drugs, but results might also be affected by factors such as the number of subjects, short treatment period, and the blood glucose levels being within the normal range.

This study has limitations as a pilot study because of the small number of subjects and the sitagliptin dose of 50 mg. A randomized, double-blind, placebo-controlled study of sitagliptin performed in Japanese patients reported that HbA1c was reduced by 0.71% after oral administration of sitagliptin 50 mg daily for 12 weeks, and by 0.69% after oral administration of sitagliptin 100 mg daily for 12 weeks, which was not a significant difference [28]. It is not known if using a dose of sitagliptin 100 mg daily in this study would have significantly affected the results. The results of larger clinical trials evaluating the cardiovascular protective effects and safety of DPP-4 inhibitors are awaited.

## Conclusion

Vildagliptin 50 mg administered twice daily to patients with type 2 diabetes mellitus significantly lowered the mean 24-h blood glucose level measured by CGM, MAGE, the highest blood glucose levels after dinner, and hyperglycemia after breakfast, compared with sitagliptin 50 mg administered once daily. There were no significant differences in BNP and PAI-1 levels between patients treated with vildagliptin and sitagliptin.

## Competing interests

The authors declare that they have no competing interests.

## Authors’ contributions

MS collected the data, performed the statistical analyses, and wrote the manuscript; RN conceived of the research hypothesis and analyses, assisted in writing the manuscript, and edited the manuscript;TI, DT and KA reviewed and edited the manuscript; KU assisted in conception of the research hypothesis and reviewed and edited the manuscript. All authors read and approved the final manuscript.

## Financial support

Financial support for this study was provided by the Japan Diabetes Foundation.
